# Platelet-rich plasma therapy in dogs with bilateral hip osteoarthritis

**DOI:** 10.1186/s12917-021-02913-x

**Published:** 2021-06-05

**Authors:** J. C. Alves, A. Santos, P. Jorge

**Affiliations:** 1 Divisão de Medicina Veterinária, Guarda Nacional Republicana (GNR), Rua Presidente Arriaga, 9, 1200-771 Lisbon, Portugal; 2 MED – Mediterranean Institute for Agriculture, Environment and Development, Instituto de Investigação e Formação Avançada, Universidade de Évora, Pólo da Mitra, Ap. 94, 7006-554 Évora, Portugal

**Keywords:** Dog, Osteoarthritis, Pain, Hip, Platelet-rich plasma, Regenerative therapy

## Abstract

**Background:**

Osteoarthritis (OA) is the most commonly diagnosed joint disease in companion animals, and hip OA is commonly diagnosed in the canine population. The use of platelet-rich plasma has gained increasing interest for the treatment of musculoskeletal conditions in companion animals. To evaluate the effect of the intra-articular administration of platelet-rich plasma in police working dogs with bilateral hip OA compared to a control group, twenty dogs were assigned to a control group (CG, *n* = 10) or treatment group (PG, *n =* 10), using the statistical analysis software. PG received two intra-articular administrations of platelet-rich plasma, 14 days apart, while CG received an intra-articular administration of saline, in the same moments. Response to treatment was determined with the Canine Brief Pain Inventory, Liverpool Osteoarthritis in Dogs, Canine Orthopedic Index, and Hudson Visual Analogue Scale, before treatment, + 8, + 15, + 30, + 60, + 90, + 120, 150, and + 180 days after initial treatment. Kaplan-Meier estimators were conducted and compared with the log-rank test. Cox proportional hazard regression analysis was performed to determine treatment survival, *p* < 0.05.

**Results:**

The sample comprised 20 animals of both sexes (male *n* = 12, female *n* = 8), with a mean age of 8.4 ± 2.4 years and a bodyweight of 31.5 ± 5.7 kg. Joints were classified as moderate (13) and severe (7) according to the Orthopedic Foundation for Animals grading scheme. No differences were found between groups at the initial evaluation. Better results with the majority of scores were observed in the PG, in some cases lasting up to the last evaluation moment. Kaplan-Meier estimators showed that PG produced longer periods with better results in all scores compared to CG. Treatment was the covariate influencing all scores in the Cox regression analysis. OFA hip score also influenced two dimensions of the Canine Orthopedic Index.

**Conclusion:**

The intra-articular administration of platelet-rich plasma can improve pain and functional scores of police working dogs with bilateral hip OA, compared with a control group. Its effects lasted for significantly longer periods, and treatment was the main covariate affecting the improvements observed.

## Background

Osteoarthritis (OA) is the most commonly diagnosed joint disease in companion animals, similarly to what happens in human medicine [1, 2]. Hip OA is commonly diagnosed in the canine population [3]. The disease leads to reduced joint function and pain, its pivotal symptom [4, 5]. There are still limited treatment options available, and the therapeutic approach focuses mainly on alleviating pain, improve function, and possibly slow down disease progression [4, 5]. For that reason, there has been a growing interest in autologous platelet therapies, as platelets may contribute to tissue regeneration, a reduction in local inflammation, and the promotion of cartilage synthesis or inhibition of its breakdown [6]. These effects are mediated by growth factors contained in platelet’s alpha granules, and include insulin-like growth factor, transforming growth factor-β, platelet-derived growth factor, vascular endothelial growth factor, and basic fibroblast growth factor [7, 8]. The efficacy of platelet-rich plasma (PRP) systems vary, as they have different compositions and characteristics regarding platelet concentration, levels of leukocytes, and red blood cells [9, 10]. The use of PRP has been described for the treatment of different musculoskeletal conditions in companion animals, both dogs [11, 12] and cats [13, 14].

OA is a complex disease and a multi-dimensional experience, including changes in limb function, in the ability to conduct daily activities, demeanor, and emotional aspect [15]. To evaluate these multiple dimensions, several clinical metrology instruments (CMI) have been developed, under a patient-centered approach [16]. Several CMIs have been submitted to criterion and construct validity testing in dogs, compared to ground reaction forces [17]. The most commonly used are the Canine Brief Pain Inventory (CBPI, divided into a pain severity score – PSS, and a pain interference score - PIS) and the Liverpool Osteoarthritis in Dogs (LOAD) [16–18]. Additional validated CMIs are the Hudson Visual Analogue Scale (HVAS), and the Canine Orthopaedic Index (COI, divided into four different dimensions, stiffness, gait, function, and quality of life – QOL) [19, 20]. Digital radiography is also a staple in the assessment of OA, and the ventrodorsal hip extended view is the most common pelvic radiographic projection used for the clinical assessment of hip OA [21].

The goal of this study was to evaluate the effectiveness of the intra-articular injection of PRP in the management of dogs with bilateral hip OA. We hypothesize that it will be able to reduce the clinical signs of OA and improve considered CMI scores, compared with a control group.

## Results

The sample included 20 police working dogs, of both genders (12 males and 8 females), with a mean age of 8.4 ± 2.4 years, bodyweight of 31.5 ± 5.7 kg, and body condition score of 4/9 (*n* = 14) and 5/9 (*n* = 6) in the Laflamme scale [22]. Four dog breeds were represented: German Shepherd Dogs (GSD, *n* = 10), Belgian Malinois Shepherd Dogs (BM, *n* = 3), Labrador Retriever (LR, *n =* 3), and Dutch Shepherd Dog (DSD, *n* = 2). Considering OFA hip grading, 13 animals were classified as moderate, and 7 as severe. All patients were followed up to the last evaluation moment (+ 180 days), and, during this period, no additional treatment or medications were administered.

**Fig. 1:**
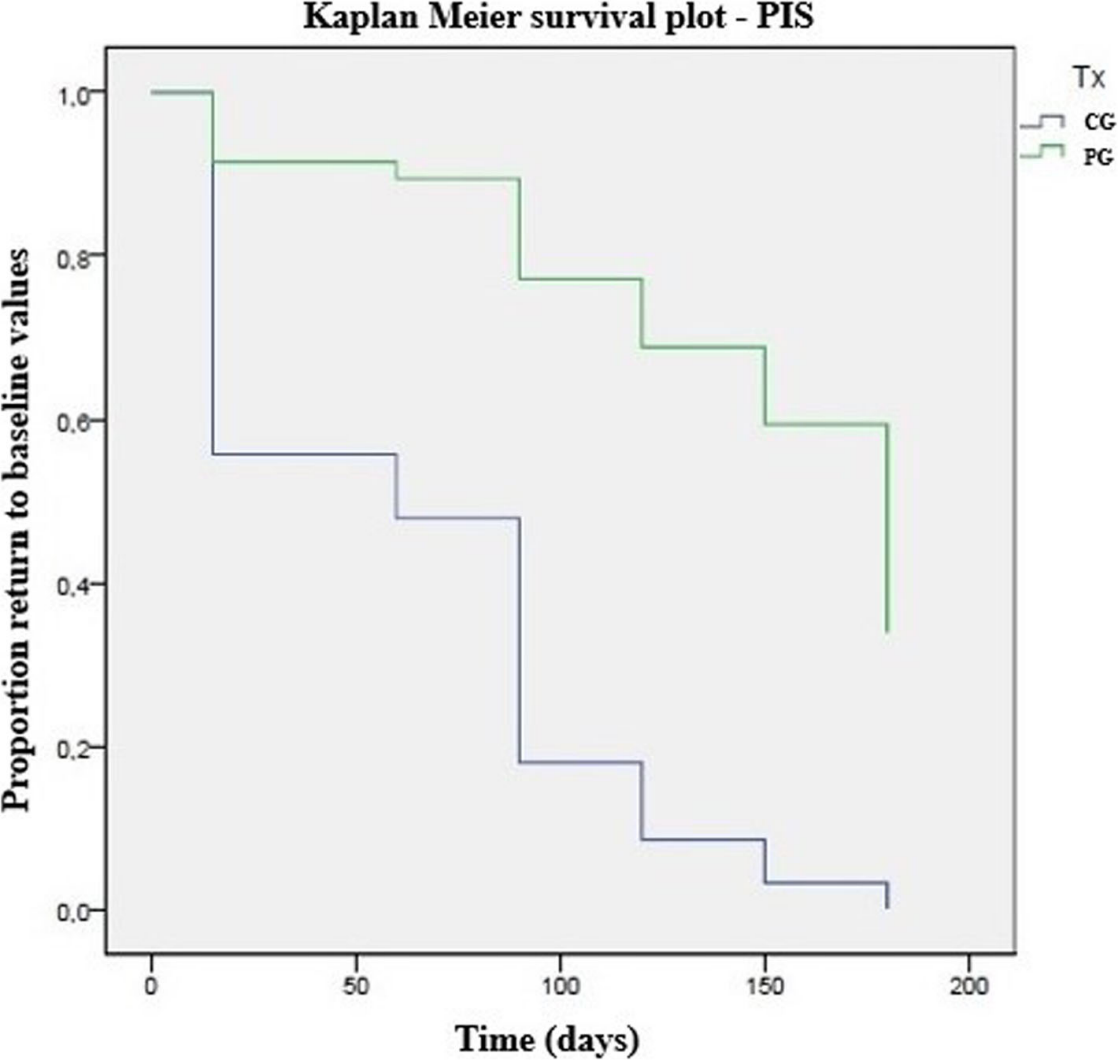
Kaplan-Meier curve demonstrating a significant difference between the control group (CG) and PRP group (PG) in time for the pain interference score (PIS) of the Canine Brief Pain Inventory to return to baseline values (*p =* 0.00)

**Fig. 2:**
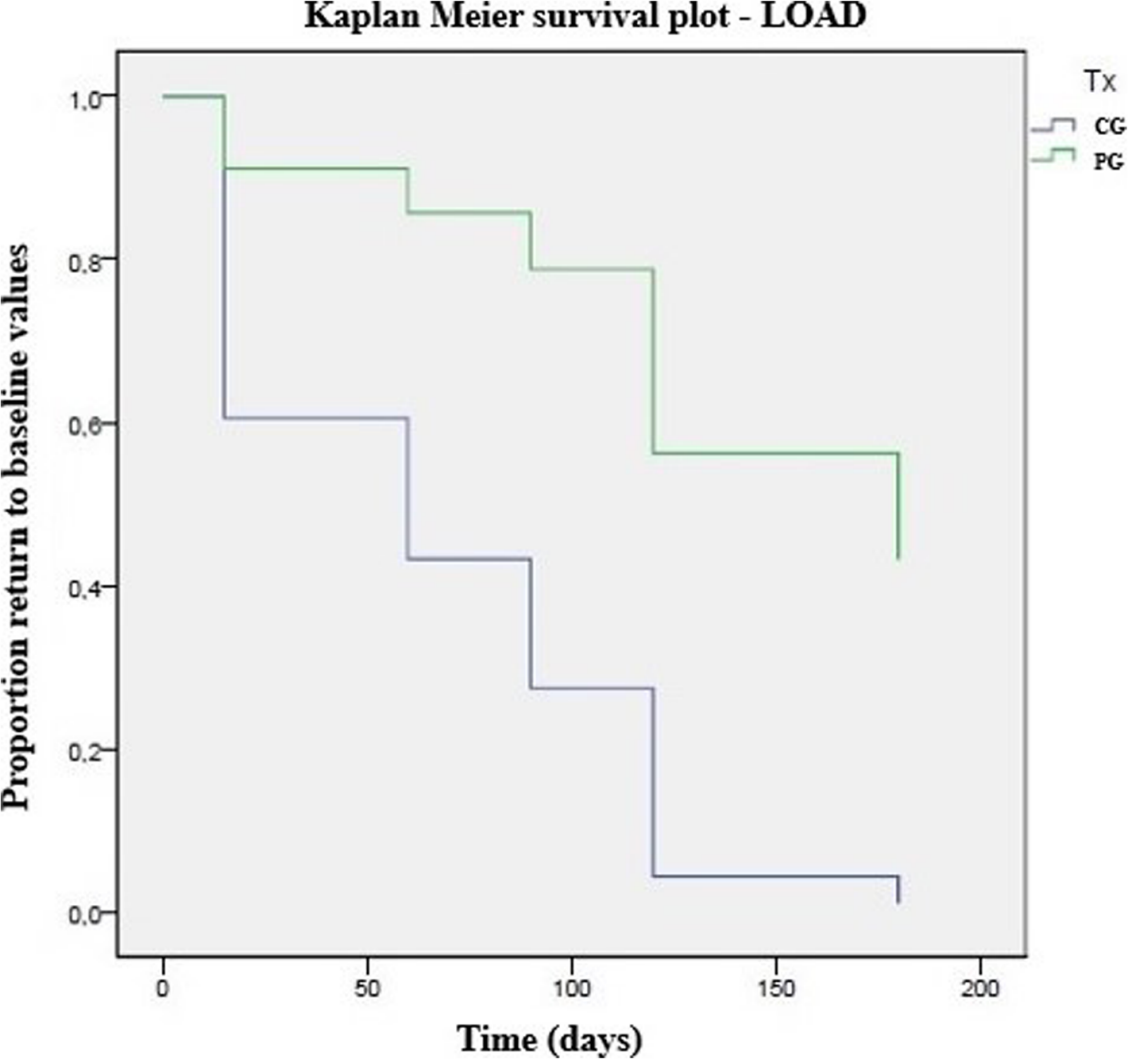
Kaplan-Meier curve demonstrating a significant difference between the control group (CG) and PRP group (PG) in time for Liverpool Osteoarthritis in Dogs (LOAD) to return to baseline values (*p =* 0.00)

The composition of the platelet concentrate and whole blood are presented in Table 1. Preparation of PRP took around 25 min from blood collection to administration. CMI scores in each group are presented in Table 2. No significant differences were observed on treatment day. Results in PG were significantly better than those in CG in several evaluation moments, particularly PSS, HVAS, and gait. Table 3 presents the results of the Kaplan-Meier estimators with each score are presented in Table 4, and Figs. 1 and 2 present Kaplan Meier plots for PIS and LOAD, respectively. PG showed more extended periods with better results, with patients taking longer to return to the initial evaluation score values. Results of the Cox proportional hazard regression are presented in Table 3. Treatment was the covariable that contributed to the outcomes observed in all scores. Increasing body weight had a 1.06-fold probability increase to baseline values. In the QOL and overall COI score, dogs with severe OA had a 2.96 and 3.02-fold increase probability, respectively, to return to baseline values compared with dogs with a moderate hip grade. Post injection increased lameness was observed in 5 patients in PG and 3 in CG, which spontaneously resolved within 48-72 h.

**Table 1:** Mean values (±standard deviation) of the composition of whole blood and platelet product

Parameter	Whole blood		Platelet concentrate	
	Mean Value	SD	Mean Value	SD
Platelets (× 10^3^/mm^3^)	299.10	84.21	1564.28	447.98
RBC (× 10^6^/mm^3^)	6.40	1.00	0.38	0.06
WDC (×10^3^/mm^3^)	10.52	4.19	8.94	3.56
Limphocytes (×10^3^/mm^3^)	2.11	0.89	4.43	1.89
Monocytes (×10^3^/mm^3^)	0.79	0.42	0.71	0.38
Neutrophils (×10^3^/mm^3^)	7.13	3.41	0.93	0.34
Eosinophils (×10^3^/mm^3^)	0.46	0.46	0.40	0.40
Basophils (×10^3^/mm^3^)	0.03	0.03	0.00	0.00

**Table 2:** Evolution of Clinical Metrology instruments, by group and moment. CBPI – Canine Brief Pain Inventory; COI – Canine Orthopedic Index; HVAS – Hudson Visual Analogue Scale; LOAD – Liverpool Osteoarthritis in Dogs; PIS – Pain Interference Score; PSS – Pain Severity Score; QOL – Quality of Life. * indicates significance when comparing groups at each follow-up moment

Clinical Metrology Instrument		Group	T0		p	+15d		p	+30d		p	+60d		p	+90d		p	+120d		p	+150d		p	+180d		p
			score	SD		score	SD		score	SD		score	SD		score	SD		score	SD		score	SD		score	SD	
**HVAS (0–10)**		CG	4.1	1.0	0.83	4.4	0.8	0.12	4.2	1.1	0.05	4.3	0.9	0.03*	4.4	1.0	0.02*	4.6	1.0	0.01*	4.8	0.8	0.02*	4.3	1.0	0.92
		PG	4.4	1.5		4.4	1.5		4.1	1.3		4.3	1.4		4.0	1.6		4.0	1.7		4.1	2.0		3.9	1.4	
**CBPI**	**PSS (0–10)**	CG	4.7	1.8	0.25	5.3	1.5	0.04*	4.4	2.1	0.01*	4.5	1.6	0.03*	4.8	1.8	0.01*	4.7	2.0	0.02*	5.2	1.3	0.04*	4.8	4.8	0.02*
		PG	6.1	1.5		4.8	2.1		4.2	2.2		4.3	2.1		4.2	2.5		4.5	2.5		4.6	2.6		4.3	1.8	
	**PIS (0–10)**	CG	5.0	1.8	0.13	5.3	1.1	0.08	4.7	2.2	0.01*	4.9	1.9	0.03*	4.8	1.8	0.01*	4.7	2.0	0.01*	5.2	1.3	0.02	4.7	1.8	0.34
		PG	6.6	1.6		5.1	2.2		4.4	2.4		4.2	2.2		4.1	2.6		4.8	2.7		4.7	2.6		4.8	2.2	
**LOAD (0–52)**		CG	19.5	6.8	0.89	23.3	5.8	0.22	26.0	8.9	0.02*	23.6	7.9	0.02*	26.2	8.7	0.02*	25.1	8.7	0.03*	22.9	8.7	0.52	22.9	8.7	0.38
		PG	24.6	8.7		22.7	8.6		22.8	8.9		22.9	10.7		23.2	11.1		23.1	10.7		23.7	10.2		20.8	8.6	
**COI**	**Stiffness (0–16)**	CG	5.8	1.9	0.22	6.9	1.9	0.04*	7.8	3.3	0.02*	7.2	2.2	0.01*	6.9	2.5	0.14	6.9	2.5	0.07	6.9	2.5	0.27	6.9	2.5	0.72
		PG	8.1	2.1		6.6	2.8		6.7	2.9		6.6	3.5		6.8	3.2		6.7	3.2		7.2	3.4		7.0	2.5	
	**Function (0–16)**	CG	7.0	2.5	0.5	8.0	3.1	0.04*	8.8	3.4	0.03*	7.5	2.5	0.01*	7.8	7.6	0.02*	7.9	3.4	0.03*	7.6	3.4	0.27	7.6	3.4	0.21
		PG	8.7	2.2		7.5	3.5		7.1	3.3		7.3	3.3		7.0	4.0		7.3	4.1		7.4	3.8		6.4	3.5	
	**Gait (0–20)**	CG	8.9	2.7	0.16	10.1	3.4	0.01*	11.2	2.9	0.03*	10.8	3.1	0.04*	10.3	3.6	0.04*	10.3	3.6	0.02*	10.2	3.6	0.04*	9.3	3.6	0.89
		PG	11.3	2.7		9.2	3.7		9.8	3.6		9.9	3.8		9.6	3.4		9.3	3.8		9.3	3.6		9.8	3.5	
	**QOL (0–12)**	CG	5.6	1.3	0.26	6.3	1.8	0.02*	6.3	2.3	0.28	6.8	2.0	0.02*	6.7	2.1	0.02*	5.8	2.1	0.62	5.8	2.1	0.62	5.8	2.1	0.47
		PG	7.5	1.9		6.1	2.0		6.2	1.9		6.1	1.9		5.8	2.6		6.2	2.5		7.0	2.7		6.4	2.1	
	**Overall (0–52)**	CG	27.3	7.8	0.16	31.3	9.87	0.01*	34.2	11.2	0.01*	31.2	8.9	0.04*	30.9	11.0	0.04*	30.9	11.0	0.04*	31.8	11.0	0.89	29.5	11.0	0.78
		PG	35.6	8.1		29.4	11.5		29.8	10.9		30.1	12.1		29.5	12.9		29.5	13.2		29.5	12.8		29.7	10.5

**Table 3:** Time (in days) to return to baseline values for CMIs, calculated with Kaplan-Meier estimators and compared with the Log-rank test. * indicates significance

Clinical Metrology Instrument		Log Rank test	Group							
			CG				PG			
			mean	SD	95% CI		mean	SD	95% CI	
CBPI	PSS	0.009*	53.1	20.2	13.4	92.7	139.5	20.3	99.8	179.2
	PIS	0.001*	35.8	9.3	17.6	53.9	136.5	18.4	100.4	172.5
HVAS		0.000*	27.7	6.97	14.02	41.4	142.5	19.6	104.1	180.9
LOAD		0.004*	43.8	12.2	19.9	67.8	131.5	18.7	94.8	168.2
COI	Stiffness	0.008*	36.9	13.0	11.4	62.4	120.0	22.7	75.6	164.4
	Function	0.046*	69.2	16.4	37.1	101.4	124.5	18.6	88.0	160.9
	Gait	0.002*	32.3	7.6	17.4	47.3	124.5	16.7	91.7	157.3
	QOL	0.003*	46.2	16.2	14.4	77.9	130.5	17.5	96.2	164.8
	Overall	0.004*	40.4	9.1	22.5	58.3	127.5	19.6	89.1	165.9

**Table 4:** Results Cox proportional hazard regression with the different outcome evaluations. BM – Belgian Malinois; CBPI – Canine Brief Pain Inventory; COI – Canine Orthopedic Index; DSD – Dutch Shepherd Dogs; GSD – German Shepherd Dogs; HVAS – Hudson Visual Analogue Scale; LOAD – Liverpool Osteoarthritis in Dogs; LR – Labrador Retriever; OFA – Orthopedic Foundation for Animals; PG – PRP group; PIS – Pain Interference Score; PSS – Pain Severity Score; QOL – Quality of Life. * indicates significance

**Variable**	**HVAS** **(*p*** = 0.001*)		**CBPI**					**LOAD** **(*p*** = 0.009*)		
			**PSS (*p*** = 0.006*)			**PIS (*p*** = 0.002*)				
	**HR (95% CI)**	**p**	**HR (95% CI)**		**p**	**HR (95% CI)**	**p**	**HR (95% CI)**	**p**	
Treatment										
PG	1.00		1.00			1.00		1.00		
Control	6.64 (1.96–22.45)	0.00*	1.82 (0.75–4.41)		0.02*	6.61 (1.97–22.19)	0.00*	5.44 (1.84–16.03)	0.002*	
OFA score										
Moderate	1.00		1.00			1.00		1.00		
Severe	1.56 (0.59–4.18)	0.37	0.86 (0.34–2.18)		0.75	0.62 (0.23–1.64)	0.33	1.08 (0.38–3.05)	0.89	
Breed		0.531								
GSD	1.00		1.00			1.00		1.00		
BM	2.02 (0.42–9.67)	0.38	0.93 (0.19–4.66)		0.93	1.46 (0.31–6.98)	0.64	2.87 (0.56–14.7)	0.21	
LR	0.63 (0.21–1.91)	0.98	0.44 (0.13–1.54)		0.98	0.58 (0.13–2.72)	0.98	0.99 (0.56–1.24)	0.98	
DSD	0.94 (0.17–5.19)	0.95	0.36 (0.06–2.33)		0.28	0.86 (0.16–4.71)	0.87	1.27 (0.24–6.89)	0.78	
Sex										
Male	1.00		1.00			1.00		1.00		
Female	0.53 (0.21–1.34)	0.18	0.50 (0.19–1.34)		0.17	0.41 (0.15–1.08)	0.07	3.07 (1.03–9.19)	0.05	
Bodyweight	1.01 (0.96–1.08)	0.65	1.06 (1.00–1.13)		0.04*	1.03 (0.97–1.10)	0.34	0.98 (0.91–1.05)	0.59	
Age	1.15 (0.92–1.45)	0.39	1.28 (0.97–1.69)		0.08	0.98 (0.79–1.22)	0.85	1.03 (0.81–1.29)	0.84	
**Variable**	**Stiffness (*p*** = 0.018*)		**Function (*p*** **= 0.178)**		**COI** Gait (p = 0.001*)		QOL (p = 0.002*)		**Total (*p*** = 0.001*)	
	**HR (95% CI)**	**p**	**HR (95% CI)**	**p**	**HR (95% CI)**	**p**	**HR (95% CI)**	**p**	**HR (95% CI)**	**p**
Treatment										
PG	1.00		1.00		1.00		1.00		1.00	
Control	4.46 (1.55–12.8)	0.01*	2.80 (1.00–7.83)	0.04*	10.72 (2.19–52.5)	0.003*	3.33 (1.21–9.22)	0.02*	3.88 (1.38–10.9)	0.01*
OFA score										
Moderate	1.00		1.00		1.00		1.00		1.00	
Severe	0.81 (0.35–1.90)	0.63	1.21 (0.47–3.09)	0.69	1.76 (0.70–4.40)	0.23	2.96 (1.15–7.62)	0.02*	3.02 (1.21–7.56)	0.02*
Breed										
GSD	1.00		1.00		1.00		1.00		1.00	
BM	1.86 (0.40–8.64	0.43	2.44 (0.53–11.16)	0.25	0.17 (0.03–0.83)	0.92	1.25 (0.26–6.08)	0.78	4.67 (0.92–23.67)	0.06
LR	1.42 (0.36–5.61)	0.98	0.46 (0.14–1.58)	0.98	0.35 (0.09–1.33)	0.99	1.39 (0.40–4.81)	0.98	1.15 (0.33–3.96)	0.98
DSD	0.89 (0.15–5.09)	0.89	1.12 (0.21–6.10)	0.89	0.76 (0.21–2.76)	0.92	0.64 (0.11–3.89)	0.63	1.73 (0.28–10.54)	0.55
Sex										
Male	1.00		1.00		1.00		1.00		1.00	
Female	0.97 (0.39–2.40)	0.94	1.14 (0.39–3.28)	0.80	0.45 (0.17–1.17)	0.10	0.29 (0.10–0.83)	0.21	0.35 (0.16–0.97)	0.04*
Bodyweight	1.01 (0.95–1.07)	0.86	0.96 (0.89–1.02)	0.20	1.01 (0.94–1.08)	0.83	1.03 (0.96–1.09)	0.39	0.99 (0.94–1.07)	0.98
Age	1.05 (0.85–1.29)	0.65	0.95 (0.77–1.18)	0.65	1.00 (0.81–1.24)	0.99	1.15 (0.92–1.44)	0.21	1.23 (0.99–1.52)	0.07

## Discussion

Osteoarthritis has a high impact on animal health and welfare, with an estimated prevalence of 200,000 dogs in the United Kingdom, and over 14 million dogs in the United States of America [1, 17]. Our results show that the IA administration of PRP can reduce pain levels and several scores of different CMIs in dogs with bilateral hip OA.

Several reports present a positive effect of IA PRP in the management of canine OA, but many are based on a surgically induced model of OA [23–25]. In dogs with naturally occurring OA, IA platelet products have shown to be able to improve clinical signs at the 12-week evaluation post-treatment [26]. IA PRP has also been able to improve clinical signs and pain levels of dogs with stifle OA due to non-stabilized cranial cruciate ligaments disease and hip dysplasia [27, 28]. Our results show that IA PRP can have a beneficial effect in police working dogs with bilateral hip OA, even in cases of severe OA. The improvement was observed in all of the considered CMI scores, in some cases starting at the first follow-up (+15d). Many scores in PG were significantly better than those of CG up to the +120d follow-up and, in the case of PSS, up to the last follow-up (+180d). A similar long-lasting effect has been described in dogs treated with PRP for the management of hip dysplasia [29], but shorter periods are presented for stifle OA [23–25]. A possible reason for this wide range of reported efficacy results of IA PRP may be related to the fact that hip OA seems to be better compensated by animals when compared to OA in other joints [30]. Also, working dogs seem to be detected at an early stage of the disease, when clinical signs and pathological changes may not be as extensive and advanced [3]. On the other hand, from our personal experience, working dogs tend to be stoic and show a higher pain tolerance compared, compared to pet dogs. An additional possibility to keep in mind is that PRP products present significant differences, even when obtained with commercially available kits, and these can be reflected in the outcomes observed [10]. When comparing different reports, the administration frequency has also to be considered, as the ideal frequency of PRP administration does not exist for dogs or humans [12, 31]. For that reason, we chose to follow the manufacturer’s recommendation. High standard deviations were observed in LOAD and overall COI, compared with the remaining CMI scores. We attribute these findings to two reasons. The first is the well-established fact that OA clinical signs do not correlate with radiographic findings and vary significantly between individuals, even with the same hip score [32]. Also, these are the CMIs with a wider range of scores (0–52 for both), while the remaining CMIs have a narrow range (0–10 for HVAS, PSS, and PIS, and 0–12, 16, or 20 in the COI dimensions).

In all considered CMIs, the mean number of days that animals in the PG took to return to the initial evaluation levels was significantly higher than in CG. Still, patients in CG did not return to their initial values at the first follow-up, as can be observed in Table 3, and Figs. 1 and 2. This effect can be attributed to a possible positive effect of the removal of the cytokine-loaded synovial fluid, followed by an administration of saline, which can further dilute the remaining cytokines within the joint space. In humans, a positive effect of saline injections has been described as lasting up to 6-months [33]. The model build with the Cox regression showed that treatment was the covariate with greater impact over the observed changes, as it had a significant difference over control in all scores. Dogs in CG always had an increased probability to return to the initial evaluation levels, varying from a 1.82 (with PSS) to 10.72-fold (with gait) probability for this event to occur. Few other covariates had an impact on the model. With PSS, increasing bodyweight corresponded to a slightly higher risk (1.06-fold). It is known that large breed and heavier dogs are more prone to develop OA earlier in life, and being overweight is a risk factor for OA [34, 35]. As the dogs included in this sample had an ideal body condition score, this increased probability can be attributed to increasing body weight. OFA grading influenced the QOL and overall COI scores, with dogs with severe OA showing a 2.96 and 3.02 probability to return to the initial values compared with dogs with moderate OA, respectively. This finding stresses the relevance of early intervention, as it leads to a better outcome. Also, the effects of PRP are expressed by interacting with the different types of joint’s cells and tissues, and in the case of severe hip OA, where significant changes have already occurred, these cells and tissues may not be as responsive or even may not be present in enough number for a more significative response to be observed [36]. Female dogs showed a lower probability to have their overall COI scores return to the initial value. This fact was not observed with any other score, and the reason for it is not clear. It may be related to the fact that male dogs may show a tendency to carry more weight on the thoracic limbs, and therefore may show fewer improvements when pelvic limbs are being treated [37].

Following intra-articular administrations, there are some side effects documented and include mainly local pain and local inflammation. These are usually self-limiting and take 2–10 days to resolve, similarly, to what we observed in both groups, with some patients showing complaints following the administration, but that resolved without external intervention during the prescribed rest period.

The study presents some limitations, namely the convenience nature of the sample and its size. All the considered CMIs have been validated, and the results of this study are reinforced by the use of several CMIs, but they can be more susceptible to bias, as is the case of the caregiver placebo effect. This effect can be present in both owners and assisting veterinarians and associated with the variability in emotional and cognitive components of OA, and with the wish for that animal to get better [38, 39]. For that reason, future studies should include an objective evaluation, as Force Plate Gait Analysis or Stance Analysis. Future studies should also evaluate the effect that different administration frequencies have on clinical results.

## Conclusions

The results of this study showed that the IA of PRP could reduce pain and improve several functional scores of police working dogs with bilateral hip OA, compared to a control group. Its effects lasted for significantly longer periods, and treatment was the main covariate affecting the improvements observed. The PRP used was safe, and no additional medications were required during the study’s follow-up period. For that reason, this PRP product can be considered as a good therapeutic option for OA management, even in more advanced cases.

## Methods

To take part in this prospective, longitudinal, double-blinded, negative controlled study, a convenience sample of 20 patients was selected. Dogs were recruited based on trainer complaints (difficulty rising, jumping, and maintaining obedience positions, stiffness and decreased overall performance), physical examination (pain during joint mobilization, stiffness, and reduced range of motion), and radiographic findings (OFA hip scores of mild, moderate or severe) consistent with bilateral hip OA. Additional inclusion criteria included age > 2 years old, bodyweight > 20 kg, and that no medications or nutritional supplements had been administered < 6 weeks. If the animal had any other confirmed or suspected orthopedic, neurologic, or concomitant disease (ruled out through physical examination, complete blood count, and serum chemistry profile), it was excluded. All animals were evaluated before beginning active training and work.

After selection, patients were randomly assigned with the statistical analysis software to two groups, a control group (CG, *n* = 10) or a treatment group (PG, *n =* 10). CG received an intra-articular (IA) administration of 2 ml of 0.9%NaCl per hip joint, while PCG received a single administration of 2 ml of PRP per hip joint, produced with the commercially available CRT PurePRP® Kit (Companion Regenerative Therapies, Newark, DE, USA), according to the manufacturer’s instructions. Briefly, 50 ml of whole blood were collected from the jugular vein of the patient, with an 18-gauge butterfly needle to a 60 ml syringe filled with 10 ml of Anticoagulant Citrate Dextrose Solution. The blood was loaded into a concentrating device and processed in the centrifuge (Executive Series Centrifuge II, Companion Regenerative Therapies, Newark, DE, USA) for 1 min at 3600 rpm. After the first centrifugation, the platelet-poor plasm and buffy coat were transferred to a second concentrating device, with a 60 ml syringe, and spun for 5 min, at 3800 rpm. A 60 ml syringe was used the remove the platelet-poor plasma, leaving 4 ml of plasma, and the concentration device was swirled to resuspend the platelets at the base. Finally, a 12 ml syringe was used to aspirate 4 ml of PRP, which was administered without activation. PRP composition was determined and compared with whole blood values. All samples were processed by an independent contract laboratory.

All IA administrations and radiographic examinations were conducted under light sedation, obtained with the simultaneous intravenous administration of medetomidine (0.01 mg/kg) and buthorphanol (0.1 mg/kg). Hips were classified according to the Orthopedic Foundation for Animals hip grading scheme at the initial evaluation, on day 0 (treatment day) [21]. The procedure for hip IA administrations has been described previously [40]. Dogs were placed in lateral recumbency with the joint to be accessed dorsal. With the greater trochanter in the center, a 4x4cm window was clipped and aseptically prepared. The limb was then placed by an assistant in a neutral position, parallel to the table, and a 21-gauge with 2.5″ length needle was then introduced just dorsal to the greater trochanter, perpendicular to the limb’s long axis until the joint was reached. In the cases where this access to the joint is not possible, probably due to bone remodeling, the assistant can externally rotate and traction the limb, thus opening up the joint. The confirmation of correct needle placement was obtained by collecting synovial fluid. If required, ultrasound guidance was available to confirm the correct needle placement. As much synovial fluid as possible was withdrawn, and the respective substance was administered. On the same day, the HVAS, CBPI, LOAD, and COI were completed sequentially by the same handler, in a quiet room with as much time as needed. According to the manufacturer’s recommendation, a second administration was performed 14 days after the first treatment. After each administration, animals were rested for three consecutive days and examined by a veterinarian on days 1 and 3 post-procedure to determine signs of exacerbated pain, persistent stiffness of gait, and posture changes. If no complaints were registered, the animal was allowed to resume its normal activity [41].

Scheduled follow-up evaluations were conducted at 15 (+15d, before the second IA PRP administration), 30 (+30d), 90 (+90d), 120 (+120d), 150 (+150d), and 180 (+180d) days after the initial treatment. At these moments, the HVAS, CBPI, LOAD, and COI were completed sequentially by the same handler. The result of previous answers was not provided to the handlers, who did not receive knowledge of their previous answers. All sections and dimensions of the CMIs were considered in the analysis. To facilitate result’s interpretation, HVAS scores were inverted by subtracting the result from 10 (the higher possible range score), since an improvement in HVAS scores consists of an increased score, while with the remaining CMIs the opposite occurs. All procedures were performed by the same researcher, blinded to the dogs’ assigned group. If the dog exhibited a decrease in performance, showed any sign of pain during exercise or manipulation, or had a decrease in the results of the CMIs, leading to a return to the initial values, it would be reevaluated as need, and rescue analgesia would be instituted.

Normality was assessed with a Shapiro-Wilk test, and results of groups in each evaluation moment were compared using a Mann–Whitney U test. Kaplan-Meier estimators were conducted to generate survival curves, survival probability and compared with the log-rank test. Cox proportional hazard regression analysis was carried out to investigate the influence of the covariables known to be of interest in OA (age, sex, body weight, breed, and OFA score) on survival. The outcome considered was a return to or drop below values recorded at the initial evaluation. With the CBPI, treatment success was defined and set as a reduction of ≥1 in PSS and ≥ 2 in PIS [42]. For that reason, with the CBPI the time for PIS and PSS scores to drop below the defined level of reduction was evaluated. Patients with values or scores above baseline values at the last evaluation moment were censored. All results were analyzed with IBM SPSS Statistics version 20, *p* < 0.05.

## Data Availability

All data generated or analyzed during this study are included in this published article.

## Abbreviations

BM: Belgian Malinois Shepherd Dog
CBPI: Canine Brief Pain Inventory
CMI: Clinical Metrology Instruments
COI: Canine Orthopedic Index
DSD: Dutch Shepherd Dog
GSD: German Shepherd Dog
HVAS: Hudson Visual Analogue Scale
LOAD: Liverpool Osteoarthritis in Dogs
LR: Labrador Retriever
OA: Osteoarthritis
PIS: Pain Interference Score
PRP: Platelet-rich plasma
PSS: Pain Severity Score
QOL: Quality of Life
